# Beyond *LKB1* Mutations in Non-Small Cell Lung Cancer: Defining LKB1less Phenotype to Optimize Patient Selection and Treatment

**DOI:** 10.3390/ph13110385

**Published:** 2020-11-13

**Authors:** Cristina Borzi, Giulia Galli, Monica Ganzinelli, Diego Signorelli, Claudio Vernieri, Marina Chiara Garassino, Gabriella Sozzi, Massimo Moro

**Affiliations:** 1 Tumor Genomics Unit, Department of Research, Fondazione IRCCS Istituto Nazionale dei Tumori, 20133 Milan, Italy; cristina.borzi@istitutotumori.mi.it (C.B.); gabriella.sozzi@istitutotumori.mi.it (G.S.); 2 Unit of Thoracic Oncology, Department of Medical Oncology, Fondazione IRCCS Istituto Nazionale dei Tumori, 20133 Milan, Italy; giulia.galli@istitutotumori.mi.it (G.G.); monica.ganzinelli@istitutotumori.mi.it (M.G.); diegosignorelli@yahoo.it (D.S.); marina.garassino@istitutotumori.mi.it (M.C.G.); 3 Niguarda Cancer Center, Grande Ospedale Metropolitano Niguarda, 20162 Milan, Italy; 4 IFOM, the FIRC Institute of Molecular Oncology, Via Adamello 16, 20139 Milan, Italy; claudio.vernieri@ifom.eu; 5 Medical Oncology Department, Fondazione IRCCS Istituto Nazionale dei Tumori, 20133 Milan, Italy

**Keywords:** NSCLC, LKB1, LKB1less phenotype, metformin, epigenetic regulation

## Abstract

LKB1 is frequently mutated in non-small cell lung cancer (NSCLC). LKB1-mutated NSCLCs often have a dismal prognosis and receive lower benefit from the currently available therapies. LKB1 acts as a cell emergency brake in low-energy conditions, by modulating the activity of crucial anabolic enzymes. Thus, loss of LKB1 activity leads to the enhancement of tumor cell proliferation also under conditions of energy shortage. This unrestrained growth may be exploited as an Achilles heel in NSCLC, i.e., by inhibiting mitochondrial respiration. Recently, clinical trials have started to investigate the efficacy of metabolism-based treatments in NSCLCs. To date, enrollment of patients within these trials is based on LKB1 loss of function status, defined by mutation in the gene or by complete absence of immunohistochemical staining. However, LKB1 impairment could be the consequence of epigenetic regulations that partially or completely abrogate protein expression. These epigenetic regulations result in LKB1 wild-type tumors with aggressiveness and vulnerabilities similar to those of LKB1-mutated ones. In this review, we introduced the definition of the “LKB1less phenotype”, and we summarized all currently known features linked to this status, in order to optimize selection and treatment of NSCLC patients with impaired LKB1 function.

## 1. Introduction

**Figure 1:**
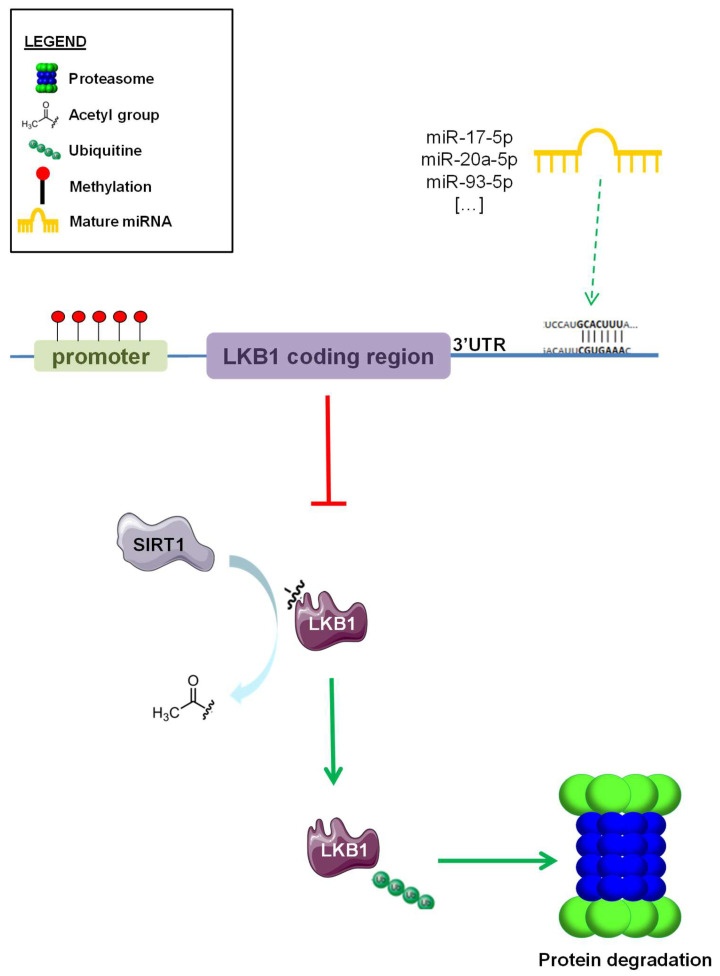
Epigenetic regulation of LKB1 expression. Different systems of epigenetic regulation of LKB1 protein expression. Promoter hypermethylation, miRNA-mediated modulation of LKB1 translation (i.e., miR-17-5p, miR-20a-5p, and miR-93-5p), and SIRT1-induced deacetylation and subsequent ubiquitination and proteasome degradation of LKB1. Green arrows indicate ubiquitination and proteasome degradation, red “T” arrow indicate inhibition of protein expression.

*STK11* (Liver Kinase B1—LKB1) is a tumor suppressor gene encoding an evolutionary conserved serine/threonine kinase. The LKB1 protein, which belongs to the calcium calmodulin family, consists of an N-terminal domain containing a nuclear localization signal and a phosphorylation site with unknown function at Serine 31, a central kinase domain, and a C-terminal domain. Intracellular localization and function of LKB1 are controlled by its interactions with STRAD and the armadillo repeat-containing mouse protein 25 (Mo25) [1]. LKB1 regulates the activity of 14 downstream kinases of the AMP-activated kinase catalytic subunit alpha 1 (AMPK) family [2]. AMPK is activated during metabolic stress, and in particular, in conditions of increased AMP/ATP and ADP/ATP ratios as a consequence of impaired glycolysis, mitochondrial metabolism, or other pathways, resulting in the inhibition of ATP biosynthesis. Once activated, AMPK catalyzes phosphorylation events that lead to the inhibition of acetyl-CoA carboxylase alpha (ACC1), 3-hydroxy-3-methylglutaryl-CoA reductase (HMGCR), and the mammalian target of rapamycin (mTOR) kinases [3], which are involved in fatty acid, cholesterol, and protein biosynthesis, respectively. By restraining the activity of these crucial anabolic enzymes, the LKB1–AMPK axis promotes energy storage and preserves intracellular ATP levels, while at the same time activating catabolic processes, such as autophagy, which lead to increased intracellular metabolic reserves. In specific conditions leading to an impairment of intracellular bioenergetics, such as cell treatment with inhibitors of mitochondrial metabolism (e.g., metformin; phenformin), the LKB1–AMPK axis can result in the activation of glucose uptake and glycolysis, which can compensate for the lack of intracellular metabolites/energetic units by promoting glucose metabolism and ATP biosynthesis. On the other hand, genetic/epigenetic inactivation of LKB1 inactivation can make cells sensitive to metformin or other inhibitors of mitochondrial respiration by restraining their ability to upregulate glucose uptake and glycolysis upon inhibition of mitochondrial metabolism [4,5,6]. In the last two decades, LKB1 has emerged as a tumor suppressor protein. Indeed, germinal mutations in the *LKB1* gene cause the Peutz–Jeghers syndrome (PJS) [7,8,9,10], an autosomal dominant inherited condition linked to an increased risk for the development of hamartomatous polyps in the digestive tract, and of melanosis. Of note, PJS patients have been reported to have a higher risk of developing pancreatic, cervical, and gastrointestinal cancers [11,12,13]. In addition, somatic *LKB1* mutations leading to LKB1 inactivation have been reported in malignant melanoma [14], cervical cancer [15], pancreatic cancer [16], breast cancer [17], and lung cancer [18]. In particular, *LKB1* is mutated in up to 30% of non-small cell lung cancers (NSCLC), and represents the third most frequently mutated gene in these tumors. Overall, more than 400 unique mutations have been described in the *LKB1* locus, with 70% of them leading to LKB1 protein truncation, while the remaining 30% represents missense mutations. Notably, many of these missense mutations lead to a loss of LKB1 oncosuppressive activity by affecting LKB1 kinase activity or localization, as well as LKB1 protein stability [19]. Besides inactivating/truncating mutations, other mechanisms may have a role in the regulation of LKB1 expression, including increased protein degradation, as described in osteosarcomas [20], or *LKB1* promoter hypermethylation [21,22,23]. Moreover, small non-coding RNA members of the miR-17~92 microRNA cluster have been reported to target the *LKB1* 3′UTR region in lymphoma models, thus leading to reduced LKB1 protein translation [24,25,26] (Figure 1). Therefore, mutation analysis of the *LKB1* gene may not be sufficient to identify patients with impaired oncosuppressive LKB1 activity.

In this paper, we review the state of the art of the impact of LKB1 inactivation on the prognosis of NSCLC patients, highlighting the peculiar and aggressive features of this subtype of NSCLC. Then, we focus on tumor and microenvironment (TME)-related characteristics which can result in impaired LKB1 activity in parallel with inactivating *LKB1* mutations. In particular, the definition of a LKB1less phenotype might be useful to improve the selection of NCSLC patients with poor clinical outcomes as a result of impaired LKB1–AMPK axis activation, and who may be candidate to receive experimental systemic treatments. Finally, we describe ongoing clinical trials aiming to target LKB1 alterations in NSCLC patients.

## 2. Role of LKB1 in NSCLC Tumor Cell Biology

LKB1 is involved in many energy-related cellular processes. Besides its “classical” role as a regulator of fatty acid, cholesterol, and protein biosynthesis, the LKB1/AMPK axis is involved in the translocation of intracellular GLUT4 to the cell surface, therefore regulating glucose uptake and modulating glycolysis [4]. Enhanced turnover of macromolecules and organelles via autophagy to produce amino acids is another process that relies on LKB1–AMPK axis activity, and which takes part in cells’ response to nutrient starvation and bioenergetic stress [27]. Moreover, LKB1 is involved in the regulation of angiogenesis by modulating the expression of genes taking part in reactive oxygen species (ROS) homeostasis, such as NADPH oxidase 1 (NOX1) [28]. The connection between LKB1 and angiogenesis is witnessed also by its role in suppressing the expression of VEGF, bFGF, MMP-2, and MMP-9 [29]. Furthermore, LKB1 loss has been linked to induction of epithelial-to-mesenchymal transition (EMT) [30] through the regulation of cell polarity and motility, and the modulation of FAK phosphorylation and CDC42 activation [31].

LKB1 has been reported to have tumor suppressor activity also in AMPK-independent ways. Indeed, LKB1 loss leads to the upregulation of the EMT transcription factor *SNAIL1*. This effect is mediated by LKB1-induced suppression of *SNAIL1* expression in a way that depends on AMPK-related kinases MARK1 and MARK4, but not on AMPK itself [32]. LKB1 has been reported to activate two other AMPK-related kinases, namely NUAK1 and NUAK2, which are involved in cell adhesion through the regulation of myosin phosphatase complexes [33]. Recently, the role of salt-inducible kinases (SIK1, 2, and 3) as downstream effectors of LKB1 has been also highlighted. The LKB1–SIK axis, which plays a key role in regulating gluconeogenesis in the liver [34], has been identified as a metastasis suppressor pathway acting as a key modulator of P53-dependent anoikis [35]. Interestingly, LKB1- and SIK-deficient lung tumors showed similar gene expression profiles, and a SIK-ness gene-expression signature has been found to be enriched in *LKB1*-mutated human lung adenocarcinomas. Moreover, the SIK-ness gene signature was reported to be regulated by LKB1 in vitro [36]. To note, the presence of characteristic patterns of gene expression seems to be a hallmark of LKB1 loss of function [37,38,39,40]. A great challenge to improve LKB1less status definition is represented by the difficulty to integrate these gene expression signatures with IHC staining of LKB1 and downstream effectors, as well as with *LKB1* mutational status.

## 3. Role of *LKB1* Mutations in the Interactions with Tumor Microenvironment (TME)

**Figure 2:**
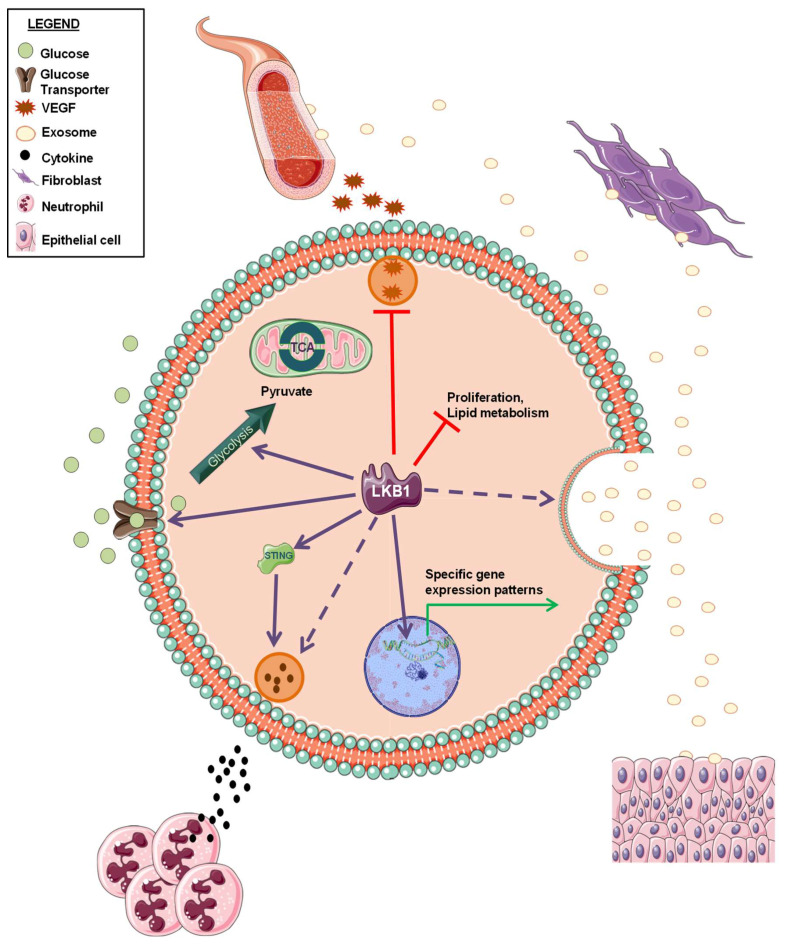
Schematic representation of LKB1 biological functions within tumor cells and in the interactions with tumor microenvironment. Solid and dashed arrows indicate a positive regulation by LKB1 (already published or not, respectively). Red “T” arrows indicate a negative regulation by LKB1.

Intelligence can be described as the ability to retain information perceived from an environment, and to apply this knowledge for adaptive behaviors. This definition seems to fit to NSCLC with impaired LKB1 function. Indeed, NSCLCs with inactive LKB1 seem to be perfectly adapted to grow in harsh conditions and to defend themselves from immune system attacks as well as from almost all therapeutic interventions. Thus, investigating the crosstalk between LKB1-inactive cancer cells and cells in their environment may offer important insights to fully characterize this subset of NSCLCs and to finally achieve a clearer definition of the LKB1less phenotype (Figure 2).

LKB1 alterations have been reported to be marker of tumor resistance to immune checkpoint blockade [41,42]. Interestingly, LKB1 loss has been related to a specific immune microenvironment, characterized by production of pro-inflammatory cytokines, a decrease in tumor-infiltrating lymphocytes and PD-L1 expression on tumor cells, and increase in neutrophils recruitment [43]. Moreover, Kitajima and colleagues demonstrated that LKB1 loss leads to the suppression of stimulator of interferon genes (STING) [44], whose activation is critical for anticancer immune response [45]. 

Whilst some studies have highlighted the role of LKB1 loss in modulating a specific immune TME, and a role of LKB1/AMPK pathway in regulating angiogenesis has been reported, very little information is available about the involvement of LKB1 in the crosstalk between tumor cells and other components in TME. A prominent component of TME is represented by cancer-associated fibroblasts (CAFs). CAFs are a heterogeneous population of cells that play a key role in regulating tumorigenesis [46]. Investigating if the metabolic changes resulting from *LKB1* loss may also affect TME cell function, including CAFs, could broaden our knowledge about the LKB1less phenotype, thus potentially suggesting new therapeutic strategies for LKB1-inactive NSCLC treatment. Interestingly, extracellular vesicles (EVs) have been also identified as players in the propagation of tumor cell signals to TME. Regarding the interaction with fibroblasts, Minciacchi et al. demonstrated that in preclinical models of prostate cancer, EV uptake induced a specific “reprogramming” of the fibroblasts towards a pro-tumorigenic phenotype [47]. Beside fibroblasts modulation, EVs’ role in metastasis formation [48] as well as in interactions between tumor cells and endothelial [49,50] or epithelial cells [51] have been described. Interestingly, recent data show that LKB1 alterations in cancer cells affects the release and the content of EVs [52,53]. However, the impact of these alterations on tumor cell–TME crosstalk has not been elucidated yet. Further investigation on the effect of LKB1 loss on EVs formation and cargo may uncover new EVs-mediated molecular mechanisms underlying the aggressiveness and malignancy of LKB1less tumors.

## 4. Evidence of LKB1 Impairment in Promoting Lung Tumorigenesis

LKB1 has well-known tumor-suppressive functions in lung cells. Indeed, an impaired function of LKB1 can promote oncogenic transformation of lung epithelial cells (LEC) to malignant cells. Interestingly, aberrant methylation of the *LKB1* promoter was more frequently detected in smokers than in non-smokers, thus suggesting a role of *LKB1* loss in smoke-induced lung cancer development [54]. Of note, *LKB1* promoter methylation correlated with worse patient overall survival (OS). Data supporting the involvement of *LKB1* inactivation in NSCLC development have been previously reported by Sasai and colleagues, who studied the in vivo capacity of Human Primary Small Airway Epithelial cells (HSAEC) to be xenografted in nude mice [55]. Interestingly, HSAECs showed enhanced in vivo tumorigenicity only when previously infected with a dominant-negative form of *LKB1* together with retroviruses expressing *KRAS*, *CDK4*, *hTERT*, and a dominant-negative form of *p53* [55]. Similar experiments carried out in human bronchial epithelial cells (HBECs) demonstrated that the overexpression of *c-MYC* greatly enhances the formation of murine malignancies, but only in the context of sh-*p53*^+^*KRAS*^V12^ HBEC [56]. Together, these data highlight the essential function of metabolic regulators, like LKB1 or c-MYC, in the malignant transformation process of lung epithelial cells (LECs). Of note, this role is appreciable only in oncogene-driven genetic backgrounds (i.e., *KRAS*-mutated cells), thus suggesting that *LKB1* loss might occur as a secondary oncogenic lesion that facilitates tumor progression in cells with one constitutively active oncogene. This hypothesis is supported by preclinical data indicating an implication of *LKB1* loss in lung cancer initiation, differentiation, and metastasis formation [57]. Indeed, in a conditionally activatable Lox-Stop-Lox KrasG12D mouse model [58], loss of LKB1 function resulted in faster tumor development, expanded histological lung cancer spectrum (adeno-, squamous, and large-cell carcinoma) and higher metastasis frequency compared to mice lacking other oncosuppressor genes like *p53* or *Ink4a/Arf* [57]. Interestingly, in these preclinical models, LKB1 loss alone did not result in tumor formation.

## 5. Clinical Significance of LKB1 Alterations in NSCLC

### 5.1. Prognostic Significance of LKB1 Status

A prognostic role of *LKB1* loss, also in a *KRAS*-mutated background, in NSCLC patients has not yet been incontrovertibly proved. Calles et al. reported that *KRAS*-mutant NSCLC patients with concurrent loss of LKB1 immunohistochemistry (IHC) expression experienced a higher number of metastatic lesions (especially extrathoracic and brain metastases) at the time of tumor diagnosis; however, no significant differences, in terms of OS, were observed in patients whose tumors lost LKB1 IHC expression [59]. Our group recently performed a post hoc analysis in advanced NSCLC patients enrolled in the multicenter, open label, randomized phase III trial TAILOR; we found that patients with *KRAS/LKB1 (KL)* co-mutated tumors did not show significantly worse OS when compared to patients with *KRAS*-mutated neoplasms [60]. However, the lack of significant decrease in patient’s overall survival (OS) may be due to the extremely short OS of stage IV NSCLC in Calles’ study and to the selection of *LKB1*-mutated, rather than IHC-negative, tumors in our series.

### 5.2. Predictive Significance of LKB1

Clinical data regarding the impact of LKB1 inactivation on the efficacy of standard NSCLC treatment mainly derive from retrospective clinical series or from subgroup analysis of prospective studies. No data from specific prospective trials are available yet.

The majority of available data indicate that LKB1 inactivation is associated with lower benefit from immunotherapy in patients with advanced NSCLC. In a pooled analysis of different cohorts of patients with *KRAS*-mutated NSCLC, including 44 patients treated in the CheckMate-057 study, Skoulidis et al. showed that LKB1-deficient NSCLC patients treated with PD-1 axis inhibitors had significantly shorter PFS and OS when compared with patients with LKB1-proficient tumors. In this study, LKB1 deficiency was defined as the presence of inactivating *LKB1* mutations, or as the lack of LKB1 protein expression at IHC analysis (H-score equal to zero). Interestingly, 17.6% of *LKB1* wild-type tumors showed absence of LKB1 protein expression by IHC, thus confirming that genetic (i.e., mutational) *LKB1* assessment might be insufficient to predict LKB1 functional status [41]. Another interesting finding of this study was the fact that *LKB1*-deficient tumors are characterized by lower PD-L1 expression, which could at least, in part, explain the low efficacy of anti-PD-1 immunotherapy agents.

In another study pulling data from several phase I/II trials, treatment with durvalumab (with or without tremelimumab) was associated with shorter patient OS and lower tumor response rates in patients with *LKB1*-mutated, advanced non-squamous NSCLC when compared to patients with *LKB1* wild-type tumors [42].

More recently, a retrospective analysis in advanced NSCLC patients treated with first-line platinum-based chemotherapy with or without pembrolizumab revealed that patients with advanced *LKB1*-mutant non-squamous NSCLC do not achieve clinical benefit from pembrolizumab addition to chemotherapy. Consistent with previously discussed results, in this study, the authors found the percentage of tumors showing strongly positive PD-L1 expression (TPS ≥ 50%) to be significantly lower in *LKB1*-mutated than in *LKB1* wild-type specimens (6.6% versus 21.7%, respectively) [61].

The negative prognostic role of LKB1 inactivation was confirmed in a large-scale, real-world, retrospective study conducted in 2407 patients with advanced NSCLC, which showed that patients with *LKB1*-mutated tumors (13.6% of the whole population) have worse PFS and OS when treated with either chemotherapy or immunotherapy, in both first- and in second-line settings [62].

Finally, in an exploratory analysis of the phase 3 MYSTIC trial, OS was shorter in patients with *LKB1*-mutated tumors when compared to patients with *LKB1* wild-type tumors, irrespective of the treatment received (i.e., immunotherapy or chemotherapy). Additionally, in this study, the authors reported a positive correlation between *LKB1* mutations and lower intratumor PD-L1 expression [63].

Together, these data consistently indicate that LKB1 inactivation in NSCLC specimens is associated with low PD-L1 expression and with lower chances to benefit from chemotherapy and/or immunotherapy in the advanced disease setting. Therefore, the possibility to avoid the use of immunotherapy in patients with LKB1-inactive NSCLC, while promoting experimental studies aimed at evaluating new treatment strategies, should be preferred in this clinical context.

To challenge this conclusion, recent data from a subgroup analysis from the phase 3 KEYNOTE-189 study showed that some patients with *LKB1*-mutated NSCLC can achieve clinical benefit from adding immunotherapy to first-line chemotherapy [64]. Even though the clinical benefit observed in this subset of patients is lower than observed in patients with LKB1 wild-type NSCLC, first-line chemo-immunotherapy remains the standard-of-care, first-line therapy for the majority of advanced LKB1-inactive NSCLC patients.

Overall, the available clinical evidence, and in data from large observational real-world genomic study and the two subgroup analyses from the phase 3 MYSTIC and KEYNOTE-189 trials, point to LKB1 inactivation as a negative prognostic factor, rather than a predictor of poor benefit from immunotherapy, in patients with advanced NSCLC.

## 6. Conclusions

Loss of oncosuppressive function of LKB1 confers peculiar characteristics to NSCLC, including clinical aggressiveness, resistance to immunotherapy, and enhanced sensitivity to metabolic stress. So far, it is not completely clear if LKB1 loss may be considered as a negative predictive factor for specific treatments, such as immunotherapy, or a negative prognostic factor tout court. However, especially when occurring together with KRAS hyperactivation, LKB1 loss is associated with dismal prognosis. Thus, broadening our knowledge on this particular subset of NSCLC is a compelling need.

**Figure 3:**
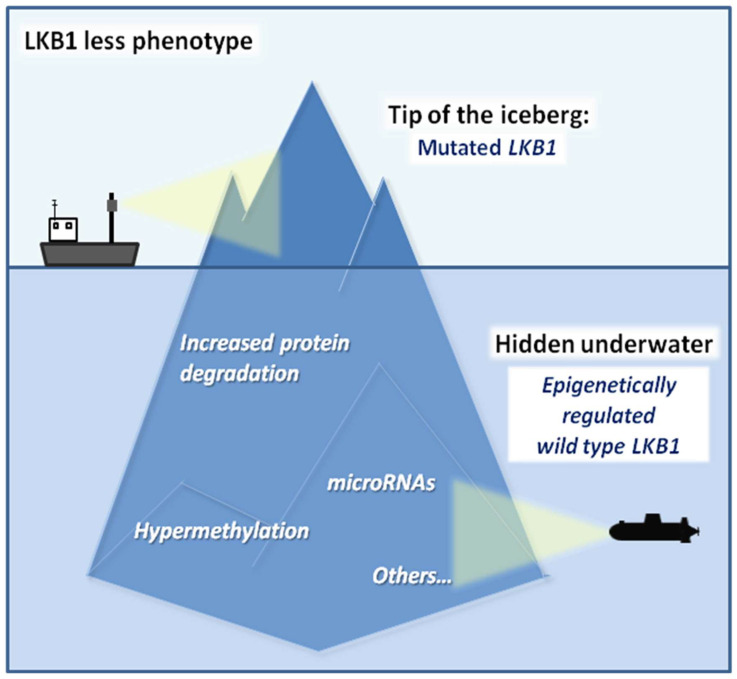
Schematic representation of the current knowledge on the LKB1less phenotype. The tip of the iceberg represents LKB1 mutations. Hidden underwater, the less investigated conditions that led to the LKB1less phenotype in wild-type LKB1 patients (hypermethylation of the promoter, non-coding RNAs, increased protein degradation, etc.) are waiting to be further investigated.

To envisage an effective therapeutic intervention for tumors with inactive LKB1, the selection of LKB1less NSCLC patients is of pivotal importance. Since LKB1 expression also depends on epigenetic mechanisms that regulate *LKB1* gene transcription, assessing *LKB1* mutational status alone may underestimate the real number of tumors with impaired LKB1 functions. In this perspective, methods capable of assessing the expression of LKB1 protein, such as IHC, are as important as *LKB1* gene sequencing to infer about the integrity of the LKB1 tumor suppressive axis. For instance, some tumors with wild-type *LKB1* gene may show low or absent LKB1 protein expression as a result of epigenetic mechanisms, impairing *LKB1* gene transcription. Conversely, missense *LKB1* mutations that impair LKB1 activity without affecting LKB1 protein expression may not be detected as loss of LKB1 staining by IHC. Based on these considerations, a better definition of the LKB1less phenotype is mandatory to refine the selection of patients who could benefit from specific treatments (Figure 3). As previously described, these LKB1less tumors show phenotypic features comparable to those of LKB1-mutated ones. Accordingly, patients with LKB1less may be considered as LKB1-mutated tumors, and may benefit from the same treatments. Therefore, an in-depth comprehension of all features causing and linked to LKB1 loss of function is mandatory to increase the survival chances of NSCLC patients with both LKB1-mutated and LKB1less tumors. 

Despite the twenty-year-old knowledge of LKB1/KRAS co-mutation in NSCLC patients, very few therapeutic interventions have been developed to specifically treat KL tumors. Only recently, some clinical trials aiming to exploit the specific vulnerabilities of KL NSCLC have been proposed. However, the current knowledge on the impact of LKB1 loss in tumor cell biology and in the interactions with TME, summarized in this review, is still too limited to allow the development of effective therapies. Thus, since “if you know yourself but not the enemy, for every victory gained you will also suffer a defeat” [65], further investigation of the causes and the implications of LKB1 loss in tumor biology is an essential point to face. Of note, no complete knowledge of our enemy, the highly aggressive KL NSCLC, may be reached without considering, both in preclinical studies and in clinical trials, the large amount of wild-type LKB1 tumors that partially or completely lost LKB1 activity: the so called “LKB1less tumors”.
